# Neck keloids: evaluation of risk factors and recommendation for keloid staging system

**DOI:** 10.12688/f1000research.9086.2

**Published:** 2016-08-19

**Authors:** Michael H. Tirgan

**Affiliations:** 1 Rockefeller University Hospital, New York, NY, 10065, USA; 2 Keloid Research Foundation, New York, NY, 10023, USA

**Keywords:** Keloid disorder, Neck keloids, Keloid staging

## Abstract

**Importance**: Health care providers have long struggled with recurrent and hard to treat keloids. Advancing our understanding of natural history and risk factors for development of large, very large and massive neck keloids can lead to improved treatment outcomes.

Clinical staging system for the categorization of keloid lesions, as well as grouping of keloid patients according to the extent of skin involvement is both fundamental for design and delivery of proper plan of care and an absolute necessity for methodical trial design and interpretation of the results thereof.

**Objective**: To review clinical presentation and natural history of neck keloids; to explore risk factors for development of large, very large and massive neck keloids; and to propose a clinical staging system that allows for categorization of keloid lesions by their size and grouping of keloid patients by the extent of their skin involvement.

**Setting:** This is a retrospective analysis of 82 consecutive patients with neck keloids who were seen by the author in his keloid specialty medical practice.

**Intervention**: Non-surgical treatment was offered to all patients.

**Results**: Neck-area keloids were found to have several unique characteristics. All 65 African Americans in this study had keloidal lesions elsewhere on their skin. Very large and massive neck keloids appear to be race-specific and almost exclusively seen among African Americans. Submandibular and submental skin was the most commonly involved area of the neck. Keloid removal surgery was found to be the main risk factor for development of very large and massive neck keloids.

**Conclusions and relevance**: Surgical removal of neck keloids results in wounding of the skin and triggering a pathological wound-healing response that often leads to formation of a much larger keloid.  Given the potential for greater harm from surgery, the author proposes non-surgical approach for treatment of all primary neck keloids.

Author’s attempts to properly categorize keloid lesions and to group the study subjects was hampered by the lack of a previously defined methodology. A clinical staging system is proposed to address the deficiency in grouping of keloid patients according to the size and extent of skin involvement with keloid lesions.

## Introduction

Neck-area keloids are fairly uncommon and seen most often among individuals with black skin. Inflammation and wounding of the skin, either from ingrown hair or shaving blades, are perhaps the leading triggering factors in formation of these keloids in genetically susceptible individuals. Although there are several publications about neck keloids, there is a void in medical literature about natural history or risk factors for development of large, very large and massive keloids in this anatomical region.

Keloid Disorder (KD) is an inherited ailment of wound-healing processes ^1^ characterized by highly variable clinical presentation that spans from individuals with one or very few small keloidal lesions to those with numerous and very large lesions involving large portions of their skin. To the author’s knowledge, no clinical staging system has been proposed to categorize KD patients according to the extent of their skin involvement.

Genetics of KD remains poorly understood. However, clinical observation suggests that the genetic predisposition to KD has a wide spectrum, from individuals who suffer from mild form of the disorder who in their lifetime only develop one or few slow-growing keloidal lesions, to those with very severe form of the disorder and who develop numerous large and fast-growing keloids; and of course, there are many others who fall somewhere in between these two extremes. As with most other genetic illnesses, there also exist many individuals who are simply carriers of the gene, who may never become symptomatic.

In addition to the genetics, several other factors play critical roles in clinical presentation of KD. Most importantly, there must exist an injury to the skin that would trigger an abnormal wound-healing response which leads to formation of keloidal lesions. Obviously, there is a wide spectrum to the severity and extent of skin injuries, ranging from very minor insults to the skin —from acne, piercing, or vaccination— to more severe forms of skin injury from surgery or burns. Besides genetics and skin injury, other important factors are age, race, gender, chronicity, therapeutic interventions and location of the keloidal lesions. The wide spectrum of all these factors contributes to highly variable phenotypes of KD.

Anatomically, the superior margin of the neck is, posteriorly at the level of superior nuchal line of the cranium and anteriorly at the level of the lower margin of the mandible. The inferior boundary of the neck is at the level of the suprasternal notch, the clavicle and the first rib. The skin boundaries between face, neck, and chest are irregular and hard to precisely define. For purpose of this publication and in accordance with the anatomical definitions, the keloids of the submandibular skin are grouped with other neck keloids.

## Materials and methods

This is a retrospective analysis of 82 consecutive patients with neck keloids who were seen by the author in his keloid specialty medical practice. Seventeen patients were Caucasians or Asians, and 65 patients were African Americans. Patients were grouped according to the size of their neck keloids, regardless of presence or absence of keloid lesions elsewhere. Keloidal lesions were assessed visually and divided into four categories. Table 1 summarizes characteristics of the patients within each group.

**Table 1.** Patients Characteristics.

Patients N=	82
**Asians/Caucasian**	**17**
Male	14
Female	3
**African Americans**	**65**
Male	25
Female	40
**Massive Neck Keloids**	**16**
Gender	
Male	6
Female	10
Race	
Caucasian - Asian	1
African American	15
**Very Large Neck Keloids**	**15**
Gender	
Male	9
Female	6
Race	
Caucasian - Asian	1
African American	14
**Large Neck Keloids**	**16**
Gender	
Male	8
Female	8
Race	
Caucasian - Asian	2
African American	14
**Minimal Neck Keloids**	**35**
Gender	
Male	16
Female	19
Race	
Caucasian - Asian	13
African American	22

**Figure 1.**
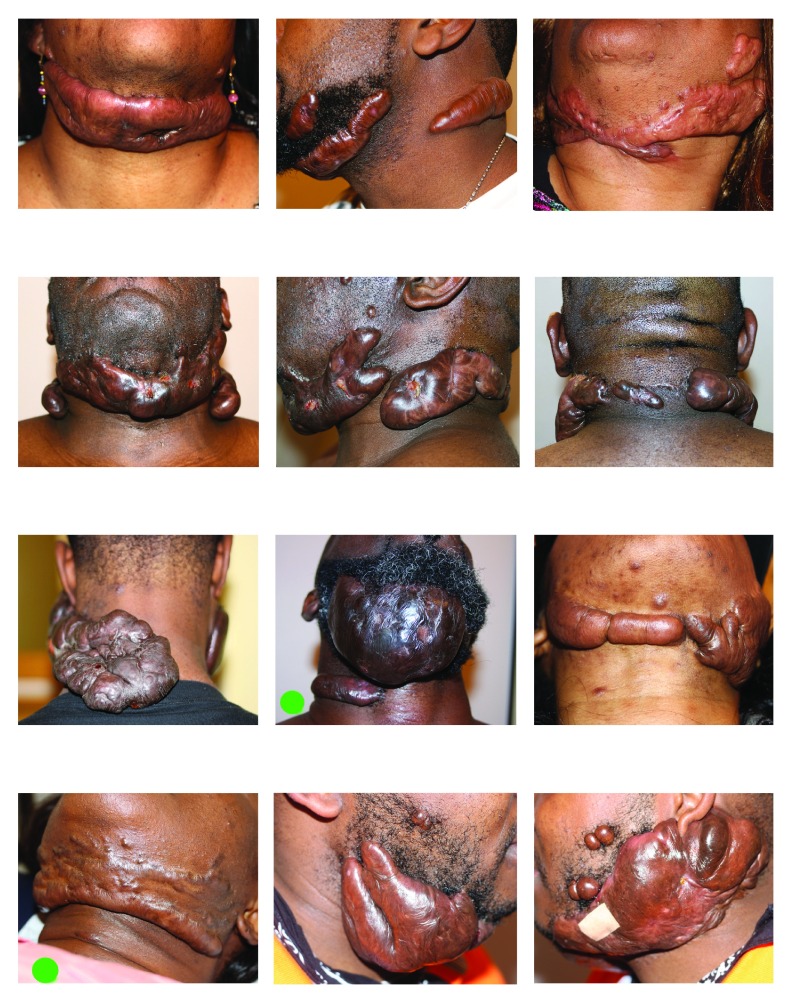
Massive neck keloids often involve both sides of the submandibular area. Green dots represent two cases of *de novo* massive keloids. All other patients have previously undergone at least one keloid-removal surgery.

**Figure 2.**
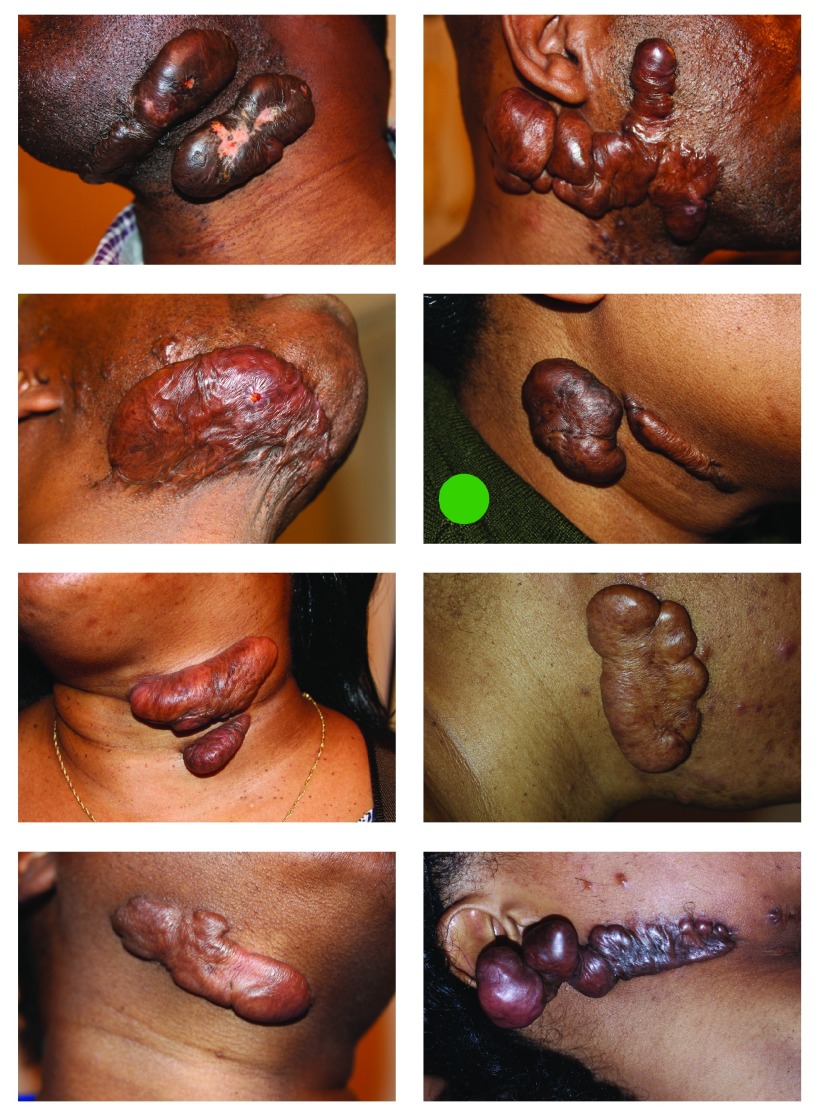
Very large neck keloids. Green dot represents one case of *de novo* massive keloids. All other patients have previously undergone at least one keloid-removal surgery.

**Figure 3.**
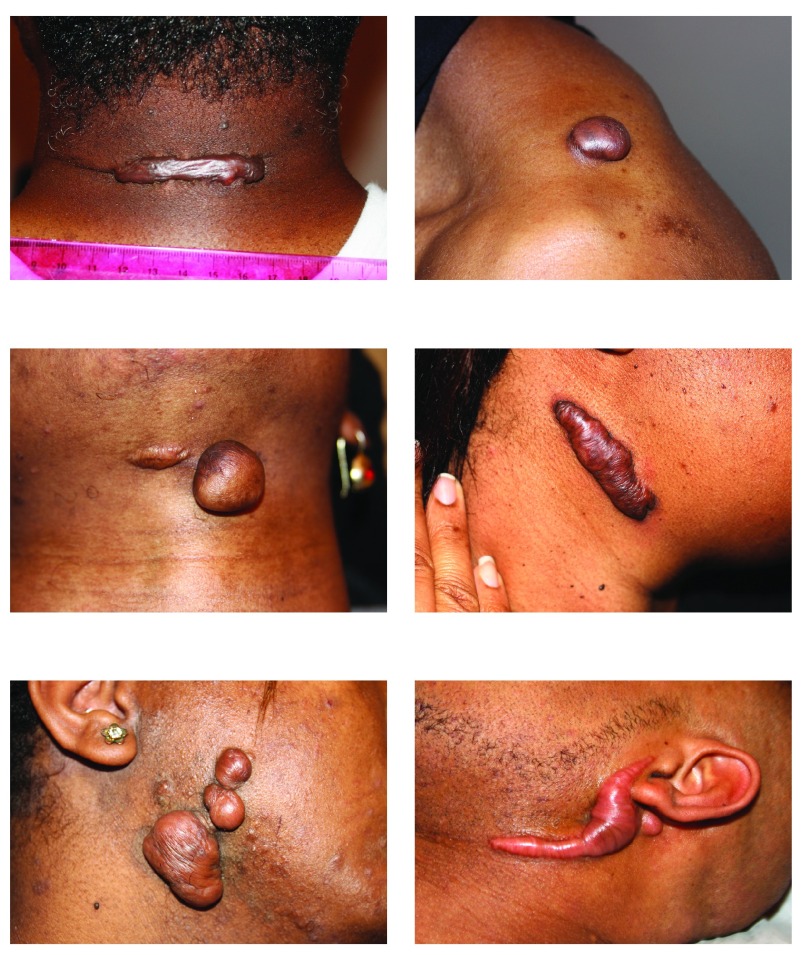
Large neck keloids presenting as tumoral lesions.

**Figure 4.**
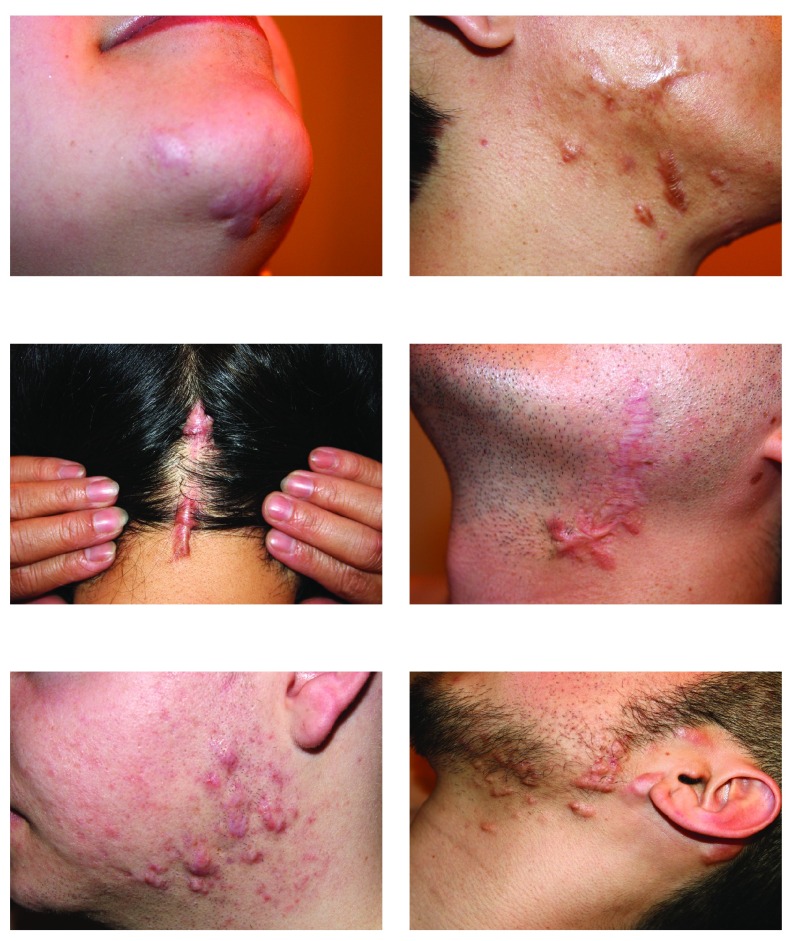
Neck Keloids lesions among Asians and Caucasians are often small in size and do not reach the tumoral sizes that are seen among African Americans.

**Figure 5.**
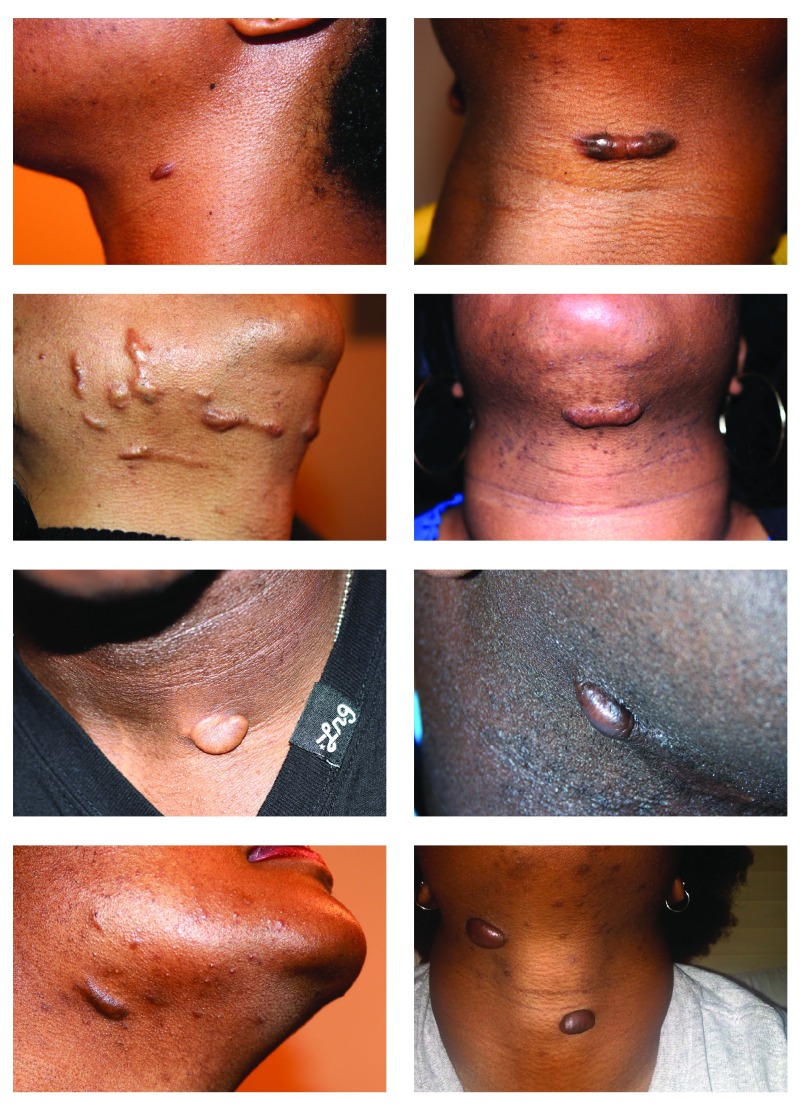
Minimal Neck Keloids lesions among African Americans.

1. **Massive** neck keloids that were very bulky and extended to both sides of the neck, often involving much of the submandibular space, or covering a very large surface of neck. Keloidal lesions of 16 patients met this criteria. Fifteen of these patients were African Americans and one was Caucasian. Figure 1 depicts some of the patients in this group.
2. **Very large** neck keloids, whereby the bulk of keloidal mass was limited to one side of the neck. Keloidal lesions of 15 patients met this criteria. Fourteen patients were African Americans and one was Caucasian. Figure 2 depicts some of patients in this group.
3. **Large** neck keloids, whereby the keloid mass formed one solitary tumor, or was comprised of multiple small lesion. Keloidal lesions of 16 patients met this criteria. Fourteen of these patients were African Americans and two were Caucasians. Figure 3 depicts some of the patients in this group.
4. **Minimal** neck keloids, whereby keloidal lesions were small in size, liner, or nodular but without formation of keloid tumors. Keloidal lesions of 35 patients met this criteria. Twenty-two patients were African Americans and 13 were Asian/Caucasian. Figure 4 depicts several of Asian or Caucasian patients. Figure 5 depicts some of the African American patients in this group.

The author was unable to find a previously described methodology, or staging system, that would allow for more precise grouping of patients with neck or other keloids. Although volumetric ultrasound measurements can be objectively applied to skin lesions ^2^, the author used visual inspection of the lesions as his sole method of grouping keloidal lesions, knowing that this method is subjective.

The IRB at Rockefeller University Hospital determined that publishing this work to be exempt from IRB review and approval process.

## Results

Despite the fact that this retrospective analysis is limited by its small sample size, several observations were made with respect to the neck-area keloids.

1. African Americans who present with neck keloids, often have keloidal lesions elsewhere in their skin. All 65 African Americans in this study had keloidal lesions elsewhere on their skin. This association was not as strong among 17 Asians and Caucasian patients, of these 11 patients had keloids elsewhere, and six patients had keloids only in their neck.
2. Neck keloidal lesions among Asians and Caucasians are usually small in size and often papular, nodular or linear; they rarely take on a tumoral form. Among all Asians and Caucasians in this study, only one patient developed massive submandibular keloid subsequent to numerus surgeries he had for removal of face and neck keloidal lesions.
3. Submandibular and submental skin was the most commonly involved area of the neck. Among 82 patients in the study, 59 patients (72%) had keloid involvement in this region of the neck.
4. Keloid removal surgery as a mean of treatment for primary neck keloids is a clear risk factor for development of very large and massive secondary neck keloids. Among 31 patients who had massive or very large neck keloids, 28 had previously undergone at least one prior keloid removal surgery.
5. Very large and massive neck keloids are race specific. There were only two Caucasians among the 31 patients with massive and very large neck keloids. Summary of this data is presented in Table 2.

**Table 2.** Results.

Patients N=	82
**African Americans**	
Patients with neck area keloids	65
Patients with keloids outside neck	65
**Asians/Caucasian**	
Patients with neck area keloids	17
Patients with keloids outside neck	11
Patients with massive neck keloids	1
**Massive and Very Large Neck Keloids**	**31**
African Americans	29
Asians/Caucasians	2
Prior keloid removal surgery	28
**Submandibular/Submental Skin Keloids**	**59**

## Discussion

Although there are numerous publications about keloids, in particular focusing on ear keloids, there is paucity of literature about neck keloids. With exception of several case reports within body of general keloid publications, there is lack of authoritative guidance, randomized studies, or even expert opinions on the proper management of neck-area keloids. PubMed search using “neck” and “keloid” as two key words does not yield any results.

**Figure 6.**
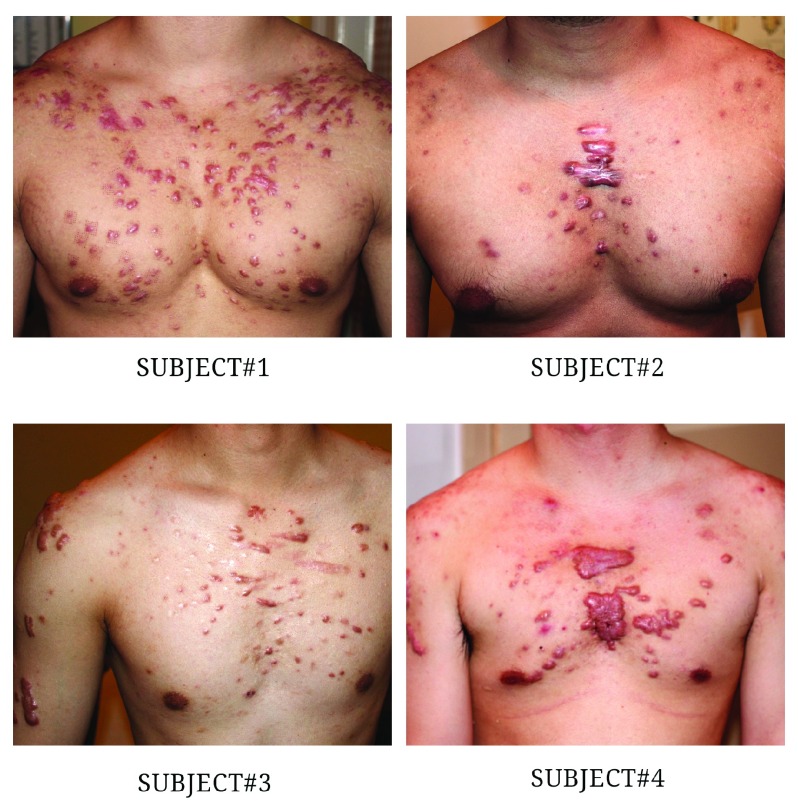
Four cases of moderate to severe chest keloids among Asian and Caucasian patients. Notice absence of neck keloids in these patients.

The skin of neck is an uncommon place for development of keloids. Indeed many KD patients, even those with the most severe form of KD, may never develop keloids in the neck-area. Figure 6 depicts four non-African American patients with moderate to severe forms of chest keloid, yet the skin of the neck in these cases seems to be spared from involvement by KD.

By far, the most important factor in the development of a primary keloidal lesion is the injury to skin that leads to triggering of a pathological wound-healing response. Although ear piercing is a well-recognized triggering factor for development of primary ear keloids, no such factor has been definitively implicated in neck keloids. Neck-area surgery is a known but uncommon triggering factor for formation of *primary* neck keloids ^3^.

Knowing that KD is a genetic disorder of wound-healing processes, it is counterintuitive to resort to surgery as the mainstay of treatment. Surgical removal of neck keloids is an intervention that is commonly practiced, not only by ear-nose-throat specialists, but also by plastic surgeons and general dermatologists. Surgical intervention however, defies the very basic principal in keloid formation. The injury and insult from surgery to the skin that surrounds a keloidal lesion, on its own, will undoubtedly trigger a keloidal wound-healing response that often leads to formation of a new keloid. Adjuvant treatments in form of post-operative steroid injections ^4^ or even radiation therapy ^5^ are commonly incorporated in management of every KD patient who undergoes surgery simply to counter the fully expected recurrence after surgery. Yet despite diligent use of all available adjuvant methods, a significant number of keloid patients undergo a second, third, or fourth surgery. In many unfortunate instances, keloids keep relapsing and at some point, either the surgeon or the patient —or both— gives up. The unfortunate patient ends up accepting the truth about inability of *surgery* to treat his or her keloids and sees no other choice but to surrender to living with huge tumoral keloids on his or her neck. Figure 1 and Figure 2 depict many such patients.

## Staging of keloid disorder

As is evident in the assessment of the size of keloid lesions and the grouping of subjects in this study, currently there is no staging system that would allow for proper categorization of keloidal lesions and meaningful grouping of keloid patients. This retrospective study is clearly handicapped by the lack of such a staging system.

TNM cancer staging system has been used for several decades and allows proper stage grouping of cancer patients ^6^. A great majority of oncology interventions, clinical trials, and standard treatments are guided by the TNM staging of the cancer at any given time. Conduct and interpretation of the result of oncology clinical trials are virtually dependent on this staging system.

Without such a staging system for KD, the interpretation of published study results is very difficult. For example, when a study looks at the rate of recurrence of ear keloids after surgery, common sense tells us that patients who only have one keloid on one ear, may have a different rate of recurrence from those who have numerous keloids on their skin. Also, those who have had prior surgical removal of their keloids will have a much higher rate of recurrence than those patients who have never had surgery before.

To assess each keloid patient properly, to better understand the natural history of this disorder, and to be able to compare future study results among various keloid patients groups, we clearly need a staging system that can allow us to describe the severity KD based on the size, location and/or extent of the keloidal lesions as well as history of surgery or radiation therapy, and perhaps other factors that are currently unknown to us. It is quite conceivable that proper management of KD patients could be guided by such a staging system. Clinical staging of KD would also be the only method that could stratify for such preexisting inherent risks of recurrence, such as response to prior treatments, positive family history, age, gender, race, and so forth.

A well-designed clinical staging system will need to be validated by review of retrospective studies as well as planned prospective clinical trials. The author hereby proposes the following staging system for KD patients.

## Clinical staging and classification of keloids

**Stage 0:**  Genetically predisposed. At least one parent has had keloids. Index person has no clinical evidence or history of keloid or any hypertrophic scars.
**Stage I:**  Presence of only one keloidal lesion.
  **Stage 1A:**  Presence of only one keloidal lesion that measures no greater than 2 centimeters in any dimension.
  **Stage 1B:**  Presence of only one keloidal lesion that measures 2.1 – 10 centimeters in any dimension.
  **Stage 1C:**  Presence of only one keloidal lesion that measures greater than 10 centimeters in any dimension.
**Stage II:**  Presence of multiple keloidal lesion. The sum of the largest diameter of the keloids is up to 30 centimeters.
  **Stage II A:**  Keloids measure ≤ 2 centimeters in largest diameter; the sum of the largest diameter of all keloids measures 10 centimeters or less.
  **Stage II B:**  Keloids measure ≤ 10 centimeters in largest diameter, at least one keloid measures 2.1 – 10 centimeters in its largest diameter; the sum of the largest diameter of all keloids measures 10.1 – 20 centimeters.
  **Stage II C:**  At least one keloid measures 10 centimeters in largest diameter; the sum of the largest diameter of all keloids measures up to 30 centimeters.
**Stage III:**  Presence of multiple keloidal lesions; the sum of the largest diameter of the keloids measures 30.1 – 50 centimeters.
  **Stage III A:**  Keloids measure ≤ 2 centimeters in largest diameter; the sum of the largest diameter of all keloids measure 30.1 – 40 centimeters.
  **Stage III B:**  Keloids measure ≤ 10 centimeters in largest diameter; at least one keloid measures 2.1 – 10 centimeters in its largest diameter; the sum of the largest diameter of all keloids measures 30.1 – 40 centimeters.
  **Stage III C:**  At least one keloid measures 10 centimeters in largest diameter; the sum of the largest diameter of all keloids measures 30.1 – 50 centimeters.
**Stage IV:**  Presence of multiple keloidal lesions; the sum of the largest diameter of the keloids is greater than 50 centimeters.
  **Stage IV A:**  Keloids measure ≤ 2 centimeters in largest diameter; the sum of the largest diameter of all keloids measures greater than 50 centimeters.
  **Stage IV B:**  Keloids measure ≤ 10 centimeters in largest diameter; at least one keloid measures 2.1 – 10 centimeters in its largest diameter. The sum of the largest diameter of all keloids measures greater than 50 centimeters.
  **Stage IV C:**  At least one keloid measures greater than 10 centimeters in its largest diameter; the sum of the largest diameter of all keloids to measure greater than 50 centimeters.

Table 3 is a summary of stage grouping of patients within each group.

**Table 3.** Proposed clinical staging system for keloid disorder.

Stage		Number of keloids	Diameter of at least one keloid (centimeters)	Sum of the largest diameter of all keloids (centimeters)
I	IA	Single	≤ 2	
	IB		2.1 – 10	
	IC		> 10	
II	IIA	Multiple	≤ 2	≤ 10
	IIB		≤ 10	10.1 – 20
	IIC		> 10	≤ 30
III	IIIA	Multiple	≤ 2	30.1 – 40
	IIIB		≤ 10	30.1 – 40
	IIIC		> 10	30.1 – 50
IV	IVA	Multiple	≤ 2	> 50
	IVB		≤ 10	> 50
	IVC		> 10	> 50
SYMP			Symptomatic	
SURG (n)			History of surgery for the index keloidal lesion(s)	
RAD			History of radiation therapy as part of keloid treatment.	

“SYMP” will designate a keloid as symptomatic. Symptoms can include pain, itching, bleeding, infection and so forth. “SURG” designation indicates to prior history of surgery for the index keloidal lesion(s). The designation “n” indicates to the number of prior surgical attempts to remove a keloid. “RAD” designation indicates to a prior history of radiation therapy for the any keloidal lesion(s) in a particular patient.

## Conclusions

Understanding the natural history of KD and recognition of risk factors that lead to formation of large, very large and massive neck keloids are the most important and fundamental elements for development of safe and effective treatment strategies. The goal of treatment for all KD patients, and those with neck-area keloids in particular, should pivot not only on removal of the keloid tissue but most importantly on prevention of the recurrence of the keloid. Performing surgery to remove primary keloidal lesions is inherently contrary to both these principles. Surgery by its nature induces a totally new injury to the skin and triggers the same dysregulated wound-healing response to a new and more extensive dermal injury which is the causal factor for formation of very large and massive neck keloids.

Systematic use of non-surgical interventions as a primary mode of treating all neck-area keloids by all health care providers will most certainly prevent the development of large, very large and incurable massive keloids. This approach will also eliminate the need for hazardous adjuvant radiation therapy.

Furthermore, a staging system for categorizing keloids and grouping of patients according to the specifics of their KD lesions will better identify the patients’ keloid-specific characteristics. Clinical staging system also has the potential to become the foundation for design and delivery of individualized plan of care. It can also become the core for methodical trial design and accurate interpretation of the study findings.

## Data availability

The data referenced by this article are under copyright with the following copyright statement: Copyright: © 2016 Tirgan MH

All raw data relevant to the study are provided in tables above.
