# Supplementary material for: Neck keloids: evaluation of risk factors and recommendation for keloid staging system
Source: F1000Res. 2016 Aug 19;5:1528. Originally published 2016 Jun 28. [Version 2] doi: 10.12688/f1000research.9086.2 (PMC5081162; doi:10.12688/f1000research.9086.2)
Supplement: Peer review history

# Peer review: Referee response for version 2

**DOI:** 10.5256/f1000research.10216.r16900

**Published:** 2016-10-11

**Authors:** Lamont R. Jones

**Affiliations:** 1 Department of Otolaryngology - Head & Neck Surgery, Henry Ford Hospital, Detroit, MI, USA

Great start to a promising staging system for neck keloids.

I have read this submission. I believe that I have an appropriate level of expertise to confirm that it is of an acceptable scientific standard.

# Peer review: Referee response for version 2

**DOI:** 10.5256/f1000research.10216.r16026

**Published:** 2016-09-01

**Authors:** Roberto Arenas

**Affiliations:** 1 Departamento of Dermatology, “Dr. Manuel Gea González” General Hospital , Tlalpan, Mexico

It is an interesting paper. The information would be better if some information about histopathological changes can be added, in order to differentiate hypertrophic scars and other fibroblastic diseases.

I have read this submission. I believe that I have an appropriate level of expertise to confirm that it is of an acceptable scientific standard.

# Peer review: Referee response for version 1

**DOI:** 10.5256/f1000research.9779.r14892

**Published:** 2016-07-18

**Authors:** Lamont R. Jones

**Affiliations:** 1 Department of Otolaryngology - Head & Neck Surgery, Henry Ford Hospital, Detroit, MI, USA

The author has attempted to address a problem that has hindered the ability to compare research and treatment outcomes for keloids. Besides a universally agreed upon marker for keloids, a staging system is paramount to successfully compare studies on keloid pathogenesis and treatment outcomes. There are some limitations to this article that should be addressed prior to publication.

1. Placing Caucasian and Asian patients into one group, although it may have been necessary in this study due to small ample size, is not optimum because of differences in risks for keloid development and treatment.
2. The grouping of keloids into massive, very large, large, and minimal neck keloids seems arbitrary and the clinical significance is unknown.
3. I agree that the conclusion that surgery for neck keloids should not be the first choice, especially for large keloids. However, information on the size of the keloids prior to the initial surgical excision is lacking and would add more validity. For example, if the keloid was 3 cm prior to initial excision and 6 cm afterwards compared to 6 cm prior to surgery and 6 cm afterwards. Furthermore, information on outcomes for non-surgical treatment options is needed.
4. Staging systems are intended to guide treatment and to predict outcomes. As presented the staging system does not satisfy either purpose. The staging system is only descriptive and its clinical implications are unknown. Larger number are needed to validate or refute the proposed staging system. 

I have read this submission. I believe that I have an appropriate level of expertise to confirm that it is of an acceptable scientific standard, however I have significant reservations, as outlined above.

# Response

**Published:** 2016-08-17

**Authors:** Michael Tirgan

**Affiliations:** Keloid Research Foundation, USA

Thank you for taking time to review and comment on my publication. I truly appreciate your detailed review, and each and every comment you have made.  I hereby address the points you have raised.

1. As for placing Asian and Caucasian patients in the same group as other patients, it was done for the following reasons: a) In all other diseases such as lung cancer, diabetes, hypertension, we do include all patients, yet we are cognizant of the biological differences that do exist among various subgroups of patients. This manuscript reviews neck area keloids, therefore all patients with neck keloids, regardless of their race, heritage or gender were included. b) I attempted to show the very interesting differences in the morphology of the disorder among races, hoping that one day we can understand the biological differences that result in a clear difference in phenotype of the disorder.  
2. As for grouping of patients in four different categories, it was done to lay the foundation that would support the proposed staging system. As you are well aware, keloid disorder is perhaps the most under-studied human ailment. We even struggle with proper terminology for it, as some still call it “keloid scarring”. As documented by numerous images, we are dealing with a semi-neoplastic process that can lead to formation of massive tumors, and yet in 2016 we have had no methods to classify, or group these patients. Therefore, the study patients were grouped according to the size of their neck keloid lesions, a methodology that is commonly used in staging tumors according to the size of the primary tumors using TNM staging system.  
3. As for the size of keloid lesions, and medical treatment outcomes, both your point are well taken as I did not report them. The focus of this manuscript was to simply classify neck keloids and show the absolute need for a staging system. The conclusions drawn, as to surgery being the dominant risk factor for massive and very large keloids, is simply supported by the data that was presented. I would love to learn about your data as to the occurrence of *de novo *massive and very large neck keloids.
4. As for staging system, I agree with all your comments. I hope that the staging system that is proposed here, however inadequate it may be, to be a starting point, so that we can all work towards improving it, or coming up with an even better one. The undisputed fact remains that we have never had a staging system for keloid disorder.   

Thank you again for all your comments.
